# Artificial Vision System on Digital Devices for Real-Time Head Tilt Control

**DOI:** 10.3390/s24123756

**Published:** 2024-06-09

**Authors:** Miguel Ángel Tomé de la Torre, Antonio Álvarez Fernández-Balbuena, Ricardo Bernárdez-Vilaboa, Daniel Vázquez Molini

**Affiliations:** 1 Optics II (Optometry and Vision), Faculty of Optics and Optometry, Complutense University, 28037 Madrid, Spain; ricardobernardezvilaboa@opt.ucm.es; 2 Optics Department, Faculty of Optics and Optometry, Complutense University, 28037 Madrid, Spain; antonioa@ucm.es (A.Á.F.-B.); dvazquez@fis.ucm.es (D.V.M.)

**Keywords:** accommodative anomalies, binocular anomalies, visio-postural control, eye tracking, image processing software, algorithm, artificial vision

## Abstract

It is common to see cases in which, when performing tasks in close vision in front of a digital screen, the posture or position of the head is not adequate, especially in young people; it is essential to have a correct posture of the head to avoid visual, muscular, or joint problems. Most of the current systems to control head inclination require an external part attached to the subject’s head. The aim of this study is the validation of a procedure that, through a detection algorithm and eye tracking, can control the correct position of the head in real time when subjects are in front of a digital device. The system only needs a digital device with a CCD receiver and downloadable software through which we can detect the inclination of the head, indicating if a bad posture is adopted due to a visual problem or simply inadequate visual–postural habits, alerting us to the postural anomaly to correct it.The system was evaluated in subjects with disparate interpupillary distances, at different working distances in front of the digital device, and at each distance, different tilt angles were evaluated. The system evaluated favorably in different lighting environments, correctly detecting the subjects’ pupils. The results showed that for most of the variables, particularly good absolute and relative reliability values were found when measuring head tilt with lower accuracy than most of the existing systems. The evaluated results have been positive, making it a considerably inexpensive and easily affordable system for all users. It is the first application capable of measuring the head tilt of the subject at their working or reading distance in real time by tracking their eyes.

## 1. Introduction

In recent years, technological advances have resulted in the overexposure of our eyes to digital screens, regardless of age, social class, or geographical area, and it is becoming increasingly common to see children at early ages with a digital device in their hands [1]. In addition to the time they devote to their education, there is an increase in the number of hours spent in front of screens and a decrease in the number of hours devoted to outdoor activities, hence the importance of good visual ergonomics when performing near-vision activities.

**Figure 1:**
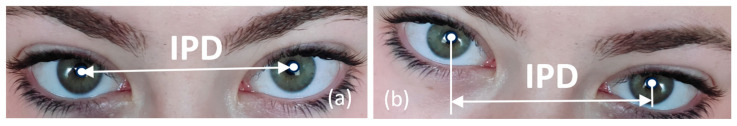
(**a**). Example of measurement of the interpupillary distance of a subject in a horizontal plane. (**b**). Reduced interpupillary distance when the head is tilted.

This overexposure to near-vision activities with an incorrect head posture can cause the appearance of visual problems, or it can be due to compensation for undiagnosed [2,3] and, therefore, uncorrected visual problems, which can lead to other muscular and joint dysfunctions. We see cases of children and adolescents in which, when they are in front of the screen of a digital device, the position of their head is not correct, tilting their head to the left or to the right, generating a decrease in the distance between pupils (IPD) with respect to the horizontal plane (Figure 1).

This decrease in IPD results in a decrease in stereo acuity, which is greater when the tilt angle increases and can affect school performance [4].

Sometimes, this torsion of the head or neck (torticollis) can arise due to non-ocular conditions [5] (musculoskeletal), for ocular reasons due to compensatory positioning, usually to maintain binocularity and/or optimize visual acuity [6], or due to a clinical condition caused by paresis of the ocular muscles, nystagmus, or torticollis of muscular origin [7].

Good visual ergonomics is fundamental, especially in the youngest patients, so being able to control their visual–postural habits is essential to be able to correct postural imbalances that can lead to muscular problems [8,9] or to diagnose a visual problem associated with inadequate posture [10].

There are devices developed to quantify head tilt; some use head-mounted motion trackers [11], and others integrated gyroscopes [12]; the one created by our team in 2016, which has two LEDs at the ends of the optical mount, is based on the same principle that we use in this application [13].

What has evolved the most are applications focused on digital devices, which are the tools that we will use the most today and in the future, both for leisure and in our work environment. In one study, the authors used a phone with an integrated camera and used comparative photos to calculate the angle of inclination [14]. There are applications that use an eye-tracking [15] system that tracks the subject’s eyes, either in mobile applications or in other digital devices, but none with the aim of measuring head tilt. We know of other applications that use facial recognition software to measure head tilt [16]. In this study, we propose the validation of a new system to control and measure the head tilt of the subjects when they are in front of a display screen, based on an iris and pupil recognition algorithm, which, through monitoring our eyes, can perform a visio-postural control of the subject’s head (tilt); in this way, we can avoid or detect possible visual complications in the future, obtaining better visual performance. Most procedures or devices require a device attached to the head to measure its inclination; others use an image recognition program, but none of them rely on eye tracking; the main advantage of our system is that it does not require any additional element attached to the head to measure the tilt; it only needs hardware with a CCD detector and downloadable software. Most people use digital devices in their daily lives, either for work, studies, or hobbies, and most of these devices already have a CCD detector, which makes it easy to control them and correct the tilt while performing their tasks; hence, this system has been proposed. The system measures the lateral tilt of the head, which is associated with various visual pathologies; in the future, we will work with new algorithms so that it can measure the different inclinations of the head. To our knowledge, we do not know of any device or application that measures head tilt in real time by checking the eyes when the subject is performing an activity in front of a digital device.

## 2. Materials and Methods

### 2.1. Design and Description of the Algorithm for Detection

For the design and creation of the new method, we need devices that use a CCD detector through which we obtain images in real time.

We developed an algorithm based on image processing techniques [17,18] that allowed us to detect and track a subject’s pupils in real time through the CCD detector.

**Figure 2:**
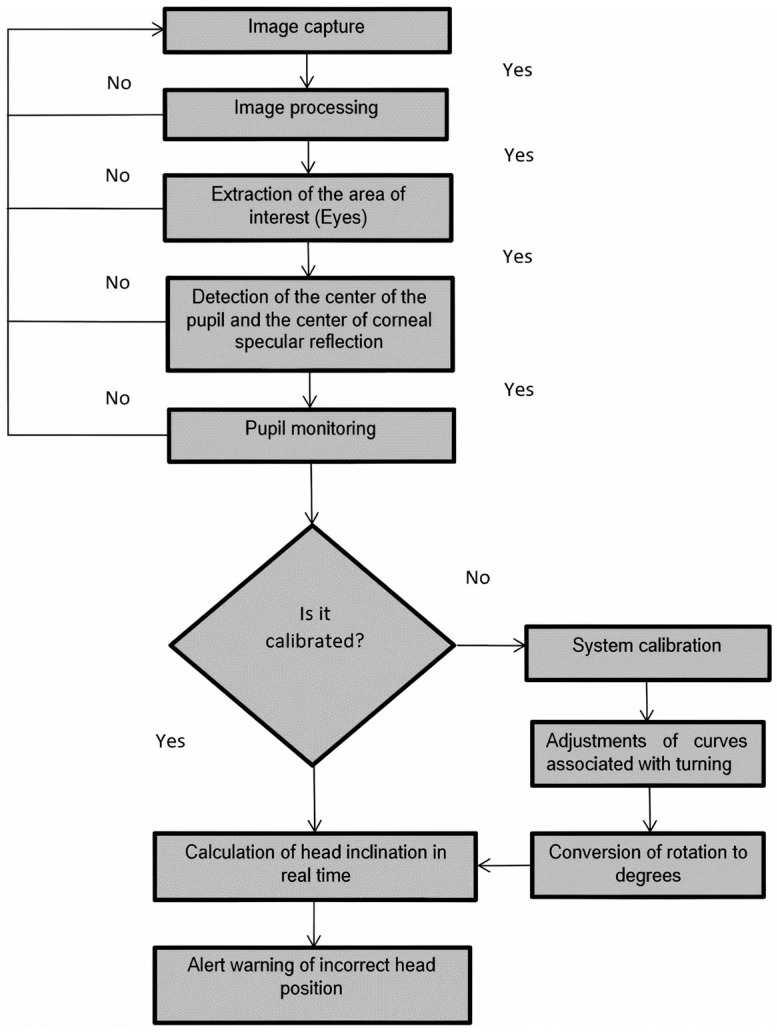
Flow chart of the system.

The calibration consists of relating the different interpupillary distances (IPDs) of the subjects to the different distances at which the task is performed. Once the method has been calibrated using the algorithm, we know the pupil coordinates, and by a simple mathematical calculation, we obtain the inclination of the subject’s head in real time, showing the degrees of inclination on the screen of the device. Figure 2 shows the flowchart of the algorithm designed to measure head tilt.

#### 2.1.1. Image Tracking and Pre-Processing Algorithm

For the creation of the algorithm, we used a well-known programming and numerical calculation platform called MathWorks [19], where we found the different algorithms for the development of our script through the MATLAB 2019 software.

##### Eye Detection

**Figure 3:**
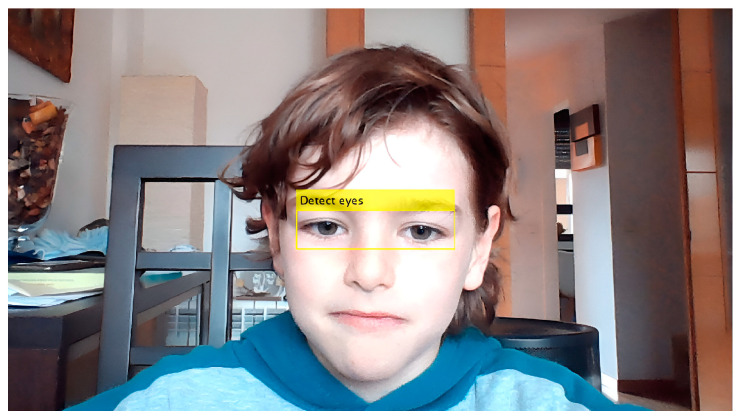
Detection of the eyes.

We captured the first frame through the CCD detector of the computer; the next step was the detection of the eyes. For the detection, we used the algorithm of Viola and Jones [20] (Figure 3), which is an object detection algorithm that uses the cascade classification ‘*vision. CascadeObjectDetector*’. The parameter chosen for detection was ‘*EyePairBig*’, which detects larger images than other models. This algorithm has a low computational cost, which allows it to be highly effective in real time and with high detection rates.

##### Eye Segmentation

**Figure 4:**
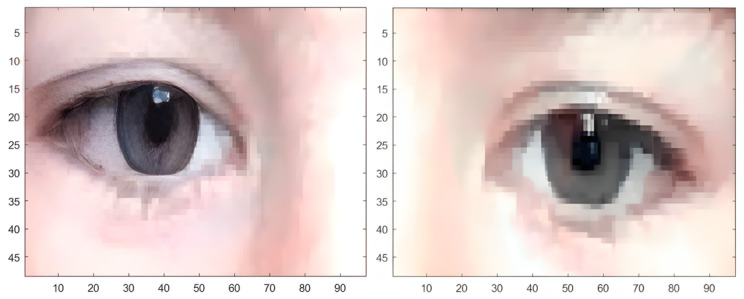
Image segmentation in half to obtain the left and right eye images separately.

Once the eyes have been detected, the next step is to segment the image using the “imcrop” command to find the iris and pupils of the subject. Once the eye area has been segmented, it is necessary to divide the image in half, separating the right eye from the left eye (Figure 4), to ease the algorithm’s detection. Once the iris and pupil are detected, the image is reconstructed to determine exactly where the coordinates are in the main image.

##### Iris Detection and Specular Reflex

**Figure 5:**
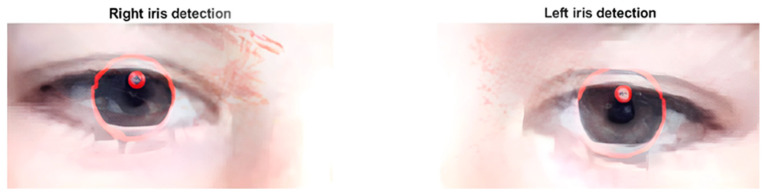
Location of the iris and center of corneal specular reflection.

For iris and pupil detection, the program ‘Iris segmentation using Daugman’s integrodifferential operator’ by Anirudh Sivaraman [21] was used, the main function being the ‘thresh’ command, returning the coordinates and centers of the iris and pupil of each eye (Figure 5).

**Figure 6:**
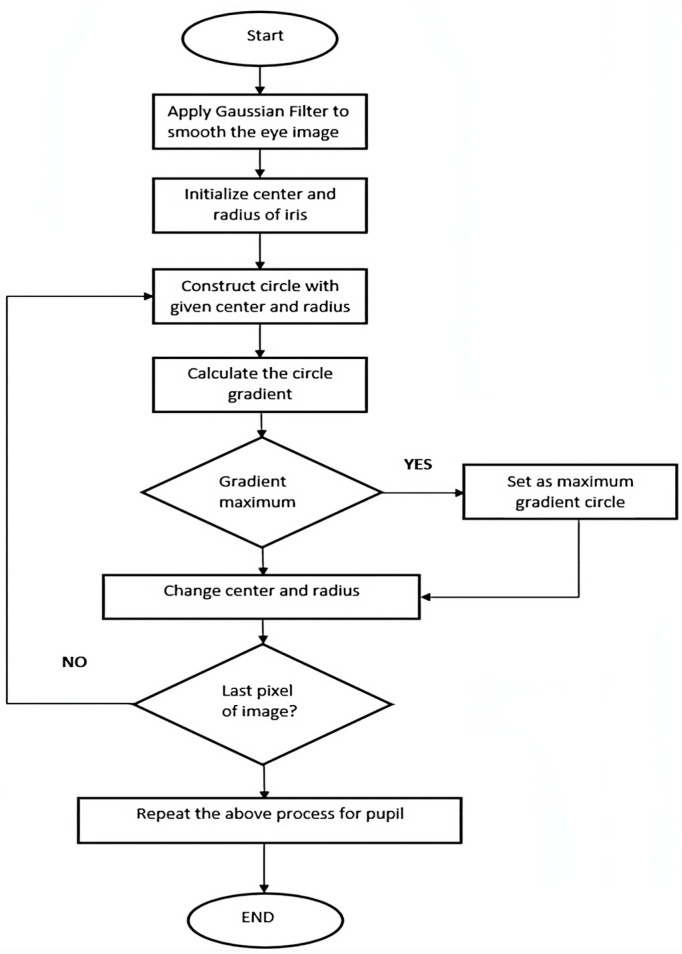
Flow chart of the Daugman’s integrodifferential operator.

The block diagram (Figure 6) and the main formula of Daugman’s integrodifferential operator [22] are as follows:

(1) $$max_{\left(r,x_{0},y_{0}\right)}\left|G_{\sigma }\left(r\right)*\frac{\partial }{\partial r\;}\oint_{r,x_{0,y_{0}}}\frac{I\left(x,y\right)}{2\pi r}\;ds\;\right|$$

I (x, y) is the intensity of the pixel at coordinates (x, y) in the image of an iris.
r denotes the radius of various circular regions with center coordinates at (x_0_, y_0_).
σ is the standard deviation of the Gaussian distribution.
Gσ(r) denotes a Gaussian filter of scale sigma (σ).
(x_0_, y_0_) are the assumed center coordinates of the iris.
s is the contour of the circle given by the parameters (r, x_0_, y_0_).

To choose the maximum and minimum radii of the iris, we chose radii that depended on the size of the box that finds the eyes since if we set fixed radii at long distances, the iris size changed and did not lead to correct detection [23].

At first, we tried the circular Hough transform model, but we saw that it was not the right method since, in real time, its computational demand was large, slowing down the process [24]. We also observed that the detection of circles at longer distances when the iris size decreased was not correct, so we did not opt for this model.

##### Iris Tracking

Once the iris was found, we checked and tracked it by point tracking using the Kanade–Lucas–Tomasi (KLT) feature-tracking algorithm [25,26]. The points to be tracked were the centers of the pupils, with the algorithm searching for them and showing them to us in real time in frames. It is possible that as the video progresses, some of the points are lost or not tracked, probably due to a variation in the illumination or the loss of fixation of the subject on the text that is presented; in this case, the application re-acquires the points again to be able to track them.

**Figure 7:**
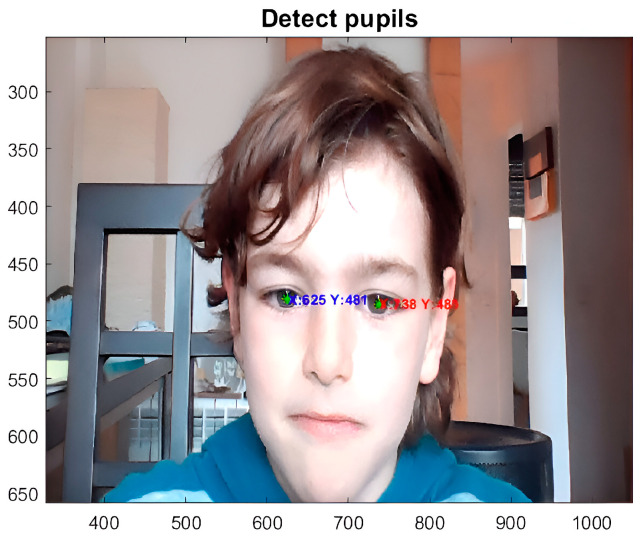
Image showing real-time eye tracking, which allows the pixel coordinates of the right and left pupillary centers to be obtained.

Once the centers of each pupil are found, we know the x- and y-pixel coordinates of the right and left pupils (Figure 7).

### 2.2. Calibration Process of the Algorithm for Head Tilt Measurement

**Figure 8:**
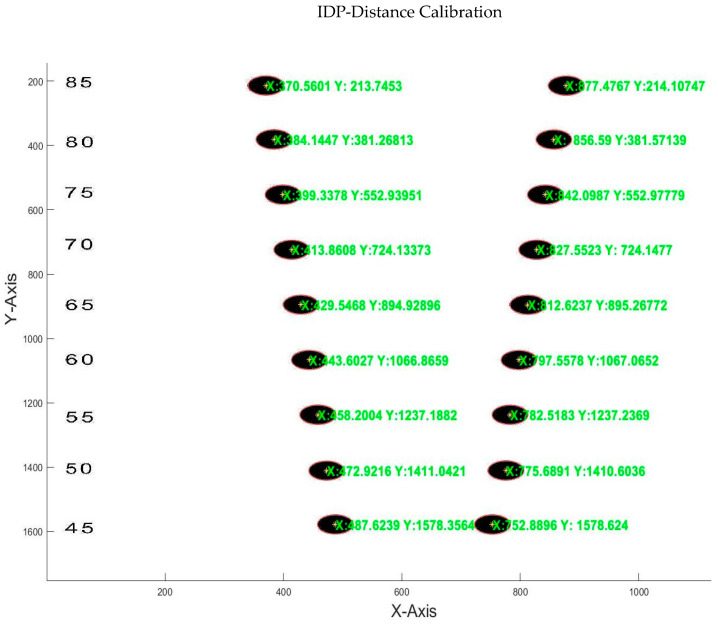
Detection of the centers of circles and their coordinates.

For tilt calibration, we created a template with a computer design program with 9 interpupillary distances with which we stood for almost all subjects [27]. IPD (Figure 8) varied between 45 and 85 mm, and was checked with a graduated ruler with a margin of error of ±1 mm.

The detection of the circles was performed with the Hough transform algorithm [28], which is a technique for shape detection by edge localization that detects the circle and the coordinates of its center.

**Figure 9:**
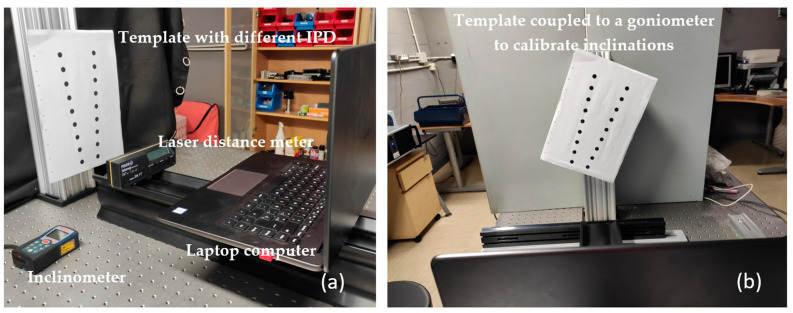
(**a**). Template with different distances between circles standing for different DIPs found in front of the laptop. (**b**). Template fixed to an optical goniometer to be calibrated at different inclinations.

To perform the calibration and measurement tests, we used a Huawei Matebook laptop computer model MRC-W60 (Figure 9a), which has an integrated camera which we used to make the video, choosing a resolution of 1280 × 720 pixels, an image frequency of 30 frames per second, and a refresh rate of 50 Hz.

The template was fixed to a goniometer with a margin of error of 0.1°, which allowed us to tilt and quantify the degrees of inclination of the points with respect to the computer camera.

The system was leveled with a Fisco model EN 17 inclinometer with a measurement accuracy of 0°/90° = +/−0.05° 1° − 89° = +/−0.2°, at 0° for both the laptop and the template.

The template was placed at different distances which were measured with a Bosch GLM40 laser rangefinder with a margin of error of ±1.5 mm, and at each distance, we measured different inclinations, taking frames of all the measurements for processing by the mathematical program.

The goniometer on which the template is fixed (Figure 9b) gives us the degrees of rotation, right in positive value and left in negative value, which we related to the separation of the pupillary centers in the vertical axis.

All calibration measurements were performed in a light environment between 200 and 400 lux.

To measure the light level, we used an RS Pro ILM 1337 lux meter with a resolution of 0.01 lux and an accuracy of ±3% + 5 dgt.

#### Adjustment by Linear Regression for the Calibration of the Inclination

**Figure 10:**
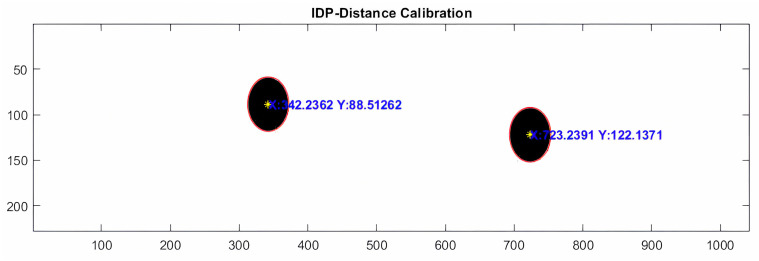
Detection of the circles when there is a rotation of the template.

We know the coordinates on the vertical (y) axis (Figure 10), so we only need to relate the degrees of tilt of the template to the vertical pixel separation of the two images (y2-y1). The result can be negative or positive depending on whether the subject tilts their head to the left or to the right.

(2) dy=y1−y2

**Figure 11:**
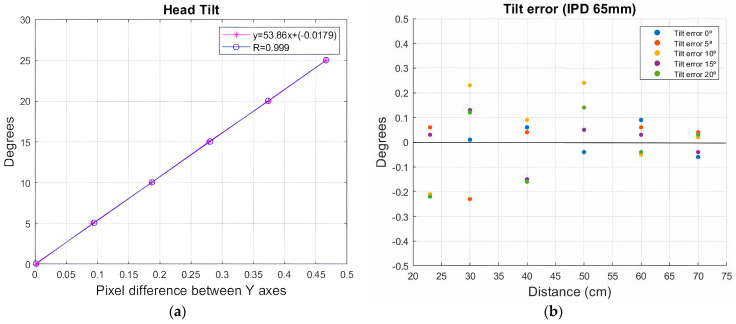
(**a**). Linear adjustment to relate the degrees and pixel difference in the Y-axis. (**b**) Error induced by the algorithm in the measurement of template inclination at different distances once calibrated for an IPD of 65 mm.

Figure 11a shows the degrees measured by a goniometer (Y-axis) and the pixel difference between the centers of the two circles (X-axis).

In this case, the equation that best relates these variables is that of a straight line, so a linear regression [29] adjustment was adopted which estimated the values of the X-axis (dependent variable) from the values of the Y-axis (independent variable), obtaining the following predictive equation:

(3) y=53.86x+(−0.0179)

The correlation coefficient R = 0.998 is close to 1, specifically 99.98% of the deviation of the Y variable with respect to its mean. The adjusted linear regression model explains this. We found that this regression model is statistically significant with a *p*-value = 1.25 × 10^−9^, ensuring that the linear model was adequate to describe the relationship that exists between these variables and predict the data.

Once the system was calibrated, five measurements were made at different inclinations, increasing by 5° at each measurement until reaching 20°. This process was performed at six different distances, starting from 23.5 cm and increasing the distance up to 70 cm. This process was repeated at different interpupillary distances, with the average measured error being less than 0.30° of inclination (Figure 11b).

### 2.3. Measurement of Head Tilt in Real Time Evaluated on Subjects

Once calibration of the system was complete, we were ready to evaluate its performance on subjects by tracking their head tilt in real time.

**Figure 12:**
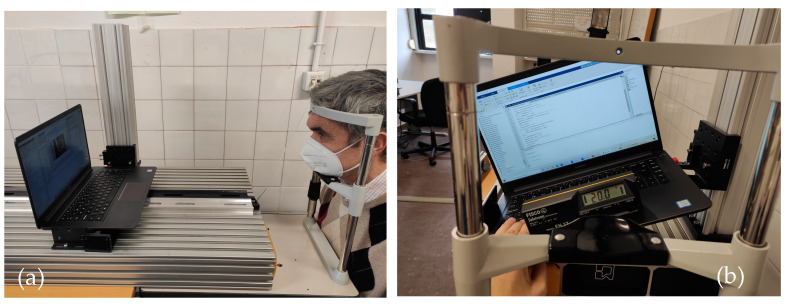
(**a**). Volunteer with his head resting on a chin rest for the start of the measurement tests. (**b**) Laptop with inclination controlled by an inclinometer to conduct the tests.

The system was evaluated on seven subjects ranging in age from 12 to 55 years. Two of the subjects, one adult and one child, were wearing their usual optical correction in their glasses at the time of testing. The test was performed on a test bench where the subject rested their head on a chin rest (Figure 12a) while being shown text on the laptop computer. The ambient light environment in which the tests were performed was 300 ± 50 lux.

The video was recorded with a computer camera with a resolution of 720 pixels, an aspect ratio of 16:9, and a speed of 30 fps.

Each subject was videotaped at different distances and at each distance at three different inclinations of the laptop computer (Figure 12b). Between 40 and 50 images were obtained from each video to determine the mean head tilt at each distance.

The first measurement was taken at the 0° inclination of the laptop, which was our starting point since the subjects did not have their eyes perfectly aligned in the horizontal plane. Later measurements subtracted the result obtained at the 0º position of the computer from the rest of the tilt measurements.

## 3. Results

**Figure 13:**
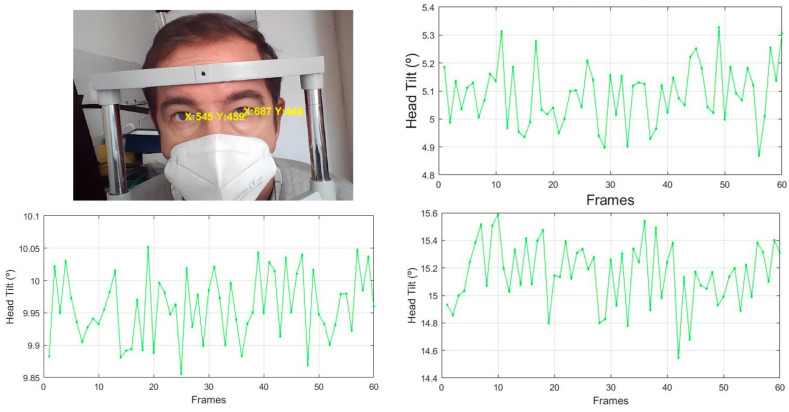
Representation of the eye tracking of the subject at 50 cm with three different inclinations at 5°, 10°, and 15°.

The system tracks the pupils by quantifying the inclination of the head in degrees with respect to the CCD receiver device in real time (Figure 13).

**Figure 14:**
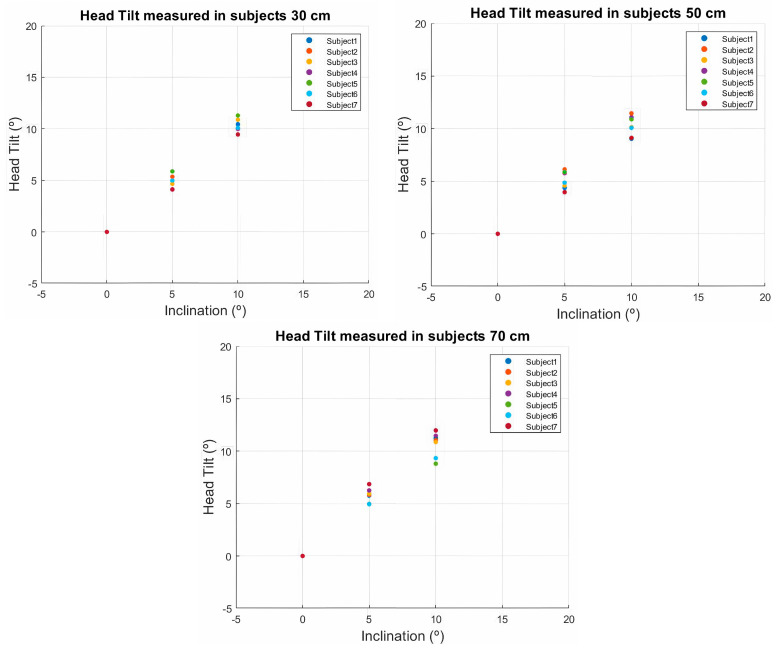
Tilt error evaluated on multiple people in real time at different distances.

The values represented in Figure 14 correspond to the different head tilt measurements performed on seven subjects. Each subject’s head tilt was tracked through a video for each tilt level to obtain the mean head tilt of each video. The process was performed at the three distances of 30, 50, and 70 cm.

For the 30 cm distance, we obtained an absolute error of 0.489 degrees for the seven subjects measured, 0.675 degrees for the 50 cm distance, and 1.070 degrees for the 70 cm distance.

**Figure 15:**
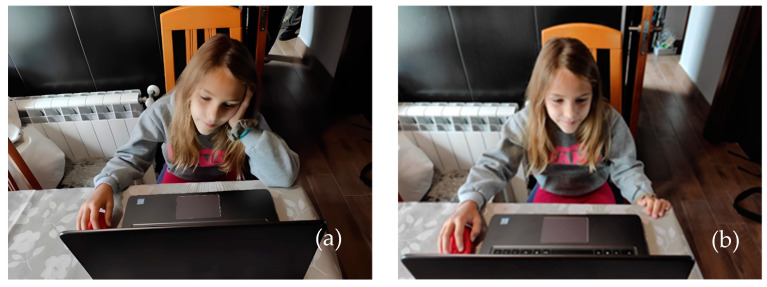

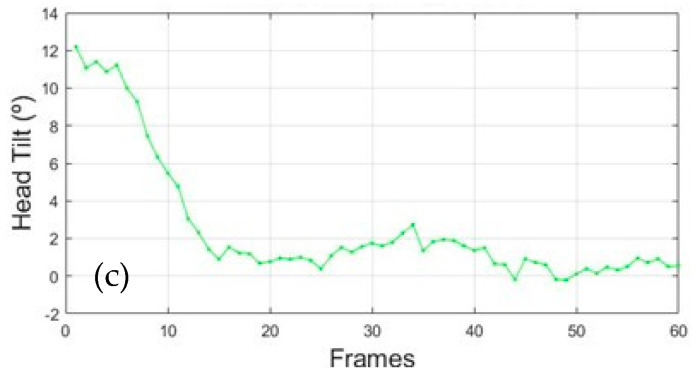
(**a**) Girl doing homework in front of the computer. (**a**) Inadequate head posture. (**b**) Correction of head posture through an audible warning system. (**c**) Representation of the degrees of head inclination.

The system was evaluated on several subjects in an open space, and the children were evaluated in their usual study environment, where the tests performed were positive. In Figure 15, we see how a girl, while performing an activity on the computer, tilts her head, adopting a posture that is not proper. The system registers her tilt in real time and warns her with a sound system until she corrects her posture.

## 4. Discussion

We can observe, especially in children and adolescents, that when they are in front of a screen, their head positioning is not correct, tilting their head to the left or to the right, which can cause muscular problems in the future or due to compensation for a visual anomaly [10].

Quantifying the head tilt of individuals in the clinical setting is not a commonly performed test, deciding the size of the tilt by observation of the patient’s head, and there is a surprisingly high degree of variability among pediatric ophthalmologists in defining standard gaze positions [30]. In some cases, the estimation of certain head tilt positions is quite inaccurate, giving an average error of 10 ± 8 degrees [31].

Several devices have been developed to check head tilt, some using analog methods, such as that of Hald et al. based on head-mounted motion trackers [11], where the repeatability limits of ninety-five percent yielded ranges of less than 10 degrees for all abnormal head postures. Others, such as that of Al-Yassary et al., carry an electronic device based on an inertial measurement unit that is attached to the subject’s head, consisting of gyroscopes and accelerometers [12], reporting the validity and reliability of the device. Unfortunately, these methods require additional devices to quantify head tilt and are not applicable in clinical practice for real-time quantification.

Recently, we found devices or methods that use facial recognition software to measure head tilt, such as the one proposed by Baek et al. [32], which uses an infrared emitter and a facial recognition algorithm, or the one proposed by Thomas et al. [15], which is very similar to our system since it runs on a standard PC with a Windows environment and uses any standard webcam for image processing, not requiring additional devices. The error increases with more extreme head posture, achieving a mean absolute error of less than 5° in the operating range of ±50° of head tilt, allowing useful quantification of the tilt in real time.

Compared to other techniques, our system does not require any added elements; it only needs hardware with a CCD detector and software that can be unloaded. Most digital devices already have this type of detector; hence, this system has been proposed.

### 4.1. Device Calibration

Calibration of the device was performed to make the system functional for all subjects with different interpupillary distances, ranging from 45 to 85 mm, and for different reading distances ranging from 23.5 cm to 70 cm. For the detection of the circles of the template, we chose a program based on the recognition of the edges of the figure; the algorithm used was the Hough transform, and we proved that it detected the circles in quite a wide range of light environments, choosing a light environment between 200 and 400 lux for the calibration, which is the light environment we found in the laboratory.

Its high correlation of 99.98% describes the relationship between these variables and the prediction of the data, ensuring good agreement between the measurement made by the system and the measurements made by the laser rangefinder.

The error produced in the calibration was not related to the different distances between the circles (DIP) or to the distance from the CCD detector to the circle template, so it did not influence the measurement of the tilt angle, obtaining an error margin of 0.30° of tilt; this error is supposed to be produced by human errors when adjusting the system and instrumental errors.

### 4.2. Measurement in Subjects

**Figure 16:**
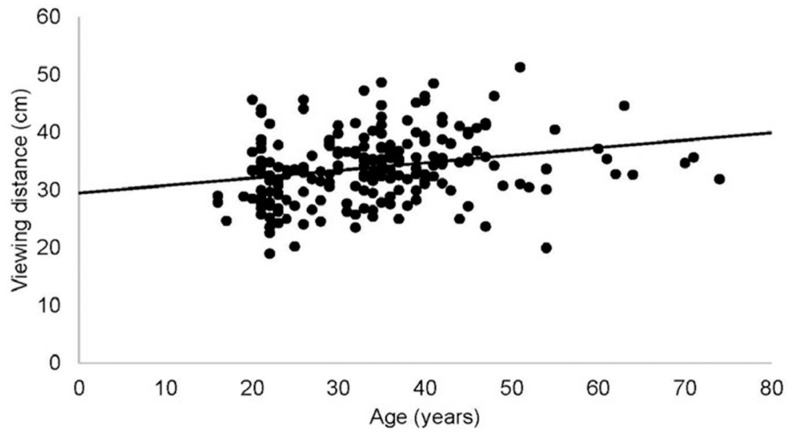
Average viewing distance of cell phones according to user age.

The results showed that in most of the variables, particularly good absolute and relative reliability values were found, measuring head inclination with an accuracy of less than 1° in the range of distances between 30 and 70 cm, which are common distances for users of this type of device. According to several studies, the viewing distance of digital devices will depend on the font size of the text [33], the age of the subject [34] (Figure 16) and viewing time [35]. 

**Figure 17:**
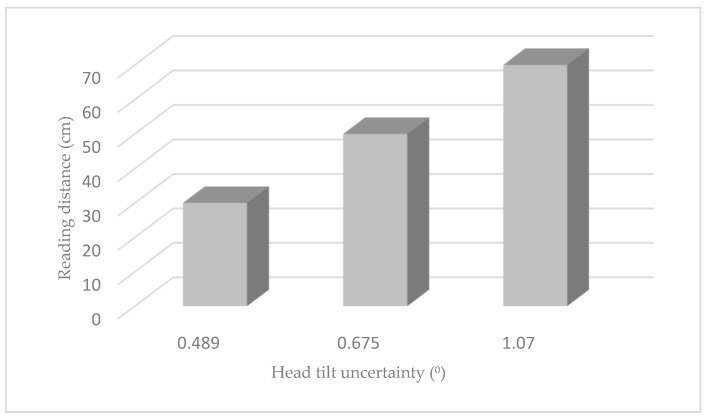
Absolute uncertainty in the measurement of head tilt at different distances.

The absolute errors achieved by our system in this validation study compared with other systems or published methods are particularly good. The average error measured in the subjects was 0.744°, increasing as we increased the distance at which the text was presented (Figure 17); this is because the pupil centers detected by the algorithm were not as accurate as at shorter distances, due to the decrease in pixels in the contour of the iris and pupil being a physical limit.

No significant differences were found in the measurement of tilt between the different interpupillary distances of the subjects. The algorithm had no problems in detecting the eyes of the subjects in different light environments and between subjects who wore optical correction (glasses) and those who did not.

The light environment of the laboratory where the measurements were taken was 300 ± 50 lux, and the system was adjusted to quantify in real time the position of the subject’s head (degrees) with a high spatiotemporal resolution, detecting the position of the subject 10 times per second. It alerts us when the position of the head exceeds a previously defined inclination by a text message and an audio warning.

To the best of our knowledge, we are not aware of any device or application that measures head tilt in real time by checking the eyes when the subject is performing an activity in front of a digital device.

Considering its high performance, ease of use, and low cost, we believe that this system has enormous potential to control head tilt and thus be able to correct and avoid visual complications in the future, making it a prevention and treatment system to achieve the best visual performance.

## 5. Conclusions

To address the postural problems of children and adults in front of visualization screens, we propose a system based on an eye-tracking algorithm using image processing to quantify and correct head tilt. The experimental results show that the system works with the different IPDs of the subjects and at different distances, measuring head tilt with an absolute error of 0.744° at a speed of 10 fps.

The system was evaluated in different lighting environments and on different subjects, with and without optical correction (glasses). The algorithm of the system perfectly detected the pupils, fulfilling the purpose of measuring the inclination of the heads of the subjects.

The evaluated results have been positive, making it a considerably affordable and easily affordable system for all users. It is the first application capable of measuring the head tilt of the subject at their working or reading distance in real time by tracking their eyes.

In the next stage of research, we will attempt to improve this algorithm so that it can also measure the distance to the display screens without the need for any additional detector, only the CCD detector, making it a more complete monitoring system when we are in front of a display screen.

## Data Availability

The data used in this study are available upon request from the corresponding author.
